# Editorial: Mental health and wellbeing of sex workers: exploring risks, resilience, and interventions

**DOI:** 10.3389/fpubh.2026.1893073

**Published:** 2026-07-02

**Authors:** Wulf Rössler

**Affiliations:** 1 Department of Psychiatry and Psychotherapy, Charité Universitätsmedizin Berlin, Berlin, Germany; 2 University of Zurich, Zurich, Switzerland

**Keywords:** mental health, meta analysis, resilience, risks, sex work, sex work and mental health

The landscape of sex work is vast and complex, encompassing millions of individuals globally, the majority of whom are women. This domain is frequently characterized by a stark dichotomy in public discourse: for some, it represents a form of agency and economic empowerment, while for others, it is inextricably linked to trauma, exploitation, and systemic violence. Regardless of the perspective, the mental health challenges faced by female sex workers (FSWs) are profound and multifaceted. Prevailing evidence indicates a significantly higher prevalence of depression, anxiety, substance use disorders, and post-traumatic stress disorder (PTSD) among sex workers compared to the general population. However, understanding these outcomes requires moving beyond simple prevalence rates to examine the intricate interplay of structural stigma, legal frameworks, working conditions, and individual resilience.

This Research Topic, “*Mental health and wellbeing of sex workers: exploring risks, resilience and interventions*,” brings together 14 articles that collectively dismantle the monolithic view of sex work. By exploring diverse geographical contexts—from Germany and the United States to low- and middle-income countries (LMICs) in Sub-Saharan Africa and Indonesia—this Research Topic highlights how social, legal, and occupational determinants shape psychological wellbeing. The contributing articles underscore that mental health outcomes are not merely a result of the sex work itself but are heavily mediated by the environment in which it occurs, the legal status of the sex workers, and the societal attitudes they encounter.

A central theme emerging from this Research Topic is the critical impact of structural and legal environments on mental health of sex workers. The legal status of sex work and the associated regulatory frameworks can either protect or endanger sex workers (Moss et al.) illuminate the devastating psychological impact of “family policing” and legal system involvement on sex-working mothers in the US, showing how surveillance and incarceration disrupt maternal identity and exacerbate mental distress. Similarly, Blouin et al. synthesize existing literature to demonstrate that mental health risks consistently increase in criminalized environments where safety and autonomy are compromised. Even in contexts where sex work is legalized, such as Germany, stigma persists. Kaya et al. reveal the “paradox of self-stigma,” where sex workers may cognitively reject negative stereotypes yet still suffer emotionally from concealment and righteous anger, leading to higher rates of affective and anxiety disorders. These findings collectively argue that legal decriminalization alone is insufficient without concomitant efforts to reduce societal stigma and protect sex workers from state-sanctioned harassment.

The heterogeneity of working settings further dictates mental health outcomes. Kalinowski et al. provide a nuanced analysis of how different work environments—ranging from street-based settings to online platforms and studios—correlate with varying levels of quality of life and psychological distress. Their work, alongside the quantitative study by Kroehn-Liedtke et al., establishes that structural stressors such as homelessness, precarious income, and lack of control over working conditions are potent predictors of mental illness. Notably, the latter study, which compares sex workers to social workers, confirms that while occupational stress is high in both fields, the specific combination of stigma and safety threats in sex work creates a unique risk profile. This emphasizes the need for setting-specific interventions rather than one-size-fits-all approaches.

Expanding the geographical scope to peripheral urban contexts, Abidjulu et al. offer a crucial social constructionist analysis of online sex work mediated by the MiChat application in Jayapura, Indonesia. Their qualitative study reveals that digital sex work is not a homogeneous phenomenon but a practice negotiated within structural constraints such as economic pressure, weak institutional support, and social stigma. Through the lenses of externalization, objectivation, and internalization, the authors demonstrate how individuals navigate multidimensional wellbeing—economic, social, psychological, spiritual, and health-related. Abidjulu et al. highlight that while digital platforms offer anonymity and flexibility, they also reproduce vulnerabilities, particularly for women who frame their involvement as a survival strategy rather than a choice. This work underscores the universality of structural inequality, showing that whether in Berlin or Jayapura, the lack of social protection and the presence of stigma are key drivers of psychological distress.

The Research Topic also sheds light on the specific vulnerabilities of subgroups within the sex worker population, particularly migrants and mothers. Migrant female sex workers (MFSWs) face compounded barriers. Lotysh et al. identify a triad of obstacles at the patient, provider, and system levels that prevent MFSWs in Germany from accessing necessary healthcare, calling for targeted strategies to bridge these gaps. The situation is even more dire in LMICs. Macias-Konstantopoulos et al. report an alarming 96.5% prevalence of probable depression among sex worker mothers in Kenya, Nigeria, and the Democratic Republic of the Congo, linking suicidal ideation directly to desperation, food insecurity, and criminalization. Furthermore, the intergenerational impact of sex work is critically examined by Weerasinghe et al., who utilize a bioecological framework to show how children of sex workers are exposed to high risks of abuse and developmental setbacks, often necessitating early entry into sex work themselves as a survival strategy. These articles collectively highlight that supporting the mental health of sex workers inevitably involves supporting their families and addressing the socio-economic determinants that trap them in cycles of vulnerability.

Trauma, both historical and contemporary, serves as another pervasive thread throughout this Research Topic. The link between Adverse Childhood Experiences (ACEs) and current mental health outcomes is robustly confirmed. Kroehn-Liedtke et al. demonstrate that severe ACEs significantly increase the odds of PTSD, depression, and substance use among women in sex work, far more so than in a control group of social care workers. This finding is echoed in the context of higher education by Lefeavers et al., who find that among college students, involvement in sex work is an independent predictor of depression, alongside recent sexual assault and childhood abuse. To understand how workers navigate this constant exposure to trauma, Sather proposes a novel theoretical framework: “persona-mediated dissociation.” This concept reframes dissociation not solely as a symptom of pathology but potentially as an adaptive, occupational strategy to compartmentalize work and self, offering a new avenue for clinical understanding and intervention.

Despite the heavy burden of risk, this Research Topic also identifies crucial resilience factors. Blouin et al. emphasize that peer and community networks function as robust protective factors, enhancing safety and access to care. Similarly, qualitative insights from Kroehn-Liedtke et al. highlight how peer support and advocacy can foster resilience even in the face of early adversity. The systematic review by Kalinowski et al. synthesizes these global findings, noting that while HIV status, migration, and substance use are significant risk amplifiers, the data also points to the urgent need for targeted, trauma-informed interventions that leverage these community strengths.

In conclusion, the 14 articles presented in this Research Topic provide a comprehensive and evidence-based call to action. They move the discourse beyond the binary of victimization vs. empowerment to a nuanced understanding of the structural, psychological, and social realities of sex work. The evidence is clear: improving the mental health of female sex workers requires more than clinical treatment. It demands policy reforms that decriminalize and protect, healthcare systems that are accessible and non-judgmental, and societal shifts that reduce stigma. As we look to the future, research must continue to explore under-represented regions and subgroups, ensuring that interventions are as diverse and complex as the population they serve. By integrating the insights from these studies, stakeholders in healthcare, policy, and social support can begin to construct a framework that truly upholds the wellbeing and dignity of female sex workers globally.

